# The Diagnostic Value of 99mTc-HMPAO-Labelled White Blood Cell Scintigraphy and 18F-FDG PET/CT in Cardiac Device-Related Infective Endocarditis—A Systematic Review

**DOI:** 10.3390/jpm11101016

**Published:** 2021-10-11

**Authors:** Katarzyna Holcman, Paweł Rubiś, Agnieszka Stępień, Katarzyna Graczyk, Piotr Podolec, Magdalena Kostkiewicz

**Affiliations:** 1 Department of Nuclear Medicine, John Paul II Hospital, 31-202 Krakow, Poland; kostkiewiczmagda@gmail.com; 2 Department of Cardiac and Vascular Diseases, John Paul II Hospital, Jagiellonian University Medical College, 31-202 Krakow, Poland; pawelrub@poczta.onet.pl (P.R.); agaa.stepien@gmail.com (A.S.); kgraczyk01@gmail.com (K.G.); ppodolec@interia.pl (P.P.)

**Keywords:** 99mTc-HMPAO-SPECT/CT, SPECT, scintigraphy, HMPAO, 18F-FDG PET/CT, infective endocarditis, CDRIE, cardiac device-related infective endocarditis

## Abstract

(1) Background: Treatment of cardiac arrhythmias and conduction disorders with the implantation of a cardiac implantable electronic device (CIED) may lead to complications. Cardiac device-related infective endocarditis (CDRIE) stands out as being one of the most challenging in terms of its diagnosis and management. Developing molecular imaging modalities may provide additional insights into CDRIE diagnosis. (2) Methods: We performed a systematic literature review to critically appraise the evidence for the diagnostic performance of the following hybrid techniques: single photon emission tomography with technetium99m-hexamethylpropyleneamine oxime–labeled autologous leukocytes (99mTc-HMPAO-SPECT/CT) and positron emission tomography with fluorodeoxyglucose (18F-FDG PET/CT). An analysis was performed in accordance with PRISMA and GRADE criteria and included articles from PubMed, Embase and Cochrane databases. (3) Results: Initially, there were 2131 records identified which had been published between 1971–2021. Finally, 18 studies were included presenting original data on the diagnostic value of 99mTc-HMPAO-SPECT/CT or 18F-FDG PET/CT in CDRIE. Analysis showed that these molecular imaging modalities provide high diagnostic accuracy and their inclusion in diagnostic criteria improves CDRIE work-up. (4) Conclusions: 99mTc-HMPAO-SPECT/CT and 18F-FDG PET/CT provide high diagnostic value in the identification of patients at risk of CDRIE and should be considered for inclusion in the CDRIE diagnostic process.

## 1. Introduction

Treatment of cardiac arrhythmias and conduction disorders with the implantation of a cardiac implantable electronic device (CIED) may lead to numerous complications [1]. Cardiac device-related infective endocarditis (CDRIE) stands out as being one of the most challenging in terms of its diagnosis and management [2]. Rising rates of CIED implantation in older patients with numerous co-morbidities has led to an increase in the prevalence of CDRIE cases [3,4]. The disease is characterized by infection extending to the electrode leads, cardiac valve leaflets or endocardial surface, typically in the form of vegetations composed of platelets, fibrin, microorganisms and inflammatory cells [2]. Due to this complex clinical presentation, currently, there is no single reliable test that can be conducted to enable diagnosis [5]. Accurate diagnosis plays a pivotal role in choosing the best treatment strategy, normally involving prolonged antibiotic therapy along with complete hardware removal [2]. The disease has severe complications and remains associated with increased in-hospital and long-term mortality; thus, developing diagnostic tools is crucial for improving the prognosis among these patients.

Developing novel molecular imaging modalities may provide additional insights into the diagnosis of CIED-associated infections and further guide tailored therapy. In recent years, there has been growing evidence supporting the application of hybrid techniques in CDRIE work-up: single photon emission tomography with technetium99m-hexamethylpropyleneamine oxime–labeled autologous leukocytes (99mTc-HMPAO-SPECT/CT) and positron emission tomography with fluorodeoxyglucose (18F-FDG PET/CT) [6]. Despite the fact that these two modalities originate in the field of nuclear medicine, they are characterized by various diagnostic properties and limitations. Moreover, the differences between them arise from the phenomena upon which these techniques are based. The first technique, 99mTc-HMPAO-SPECT/CT, relies on the intracellular labeling of leukocytes, which are isolated from whole blood with 99mTc-HMPAO complex and are intravenously administered [6,7]. The second modality, 18F-FDG PET/CT, is based on the radiolabeled glucose analog (18F-FDG). The 18F-FDG enters cells through specific glucose transporters expressed on their surface, is phosphorylated by hexokinase, becomes trapped and accumulates within activated inflammatory cells such as leukocytes, macrophages and lymphocytes. [8,9,10]. These different underlying molecular mechanisms and acquisition protocols entail the distinct properties of 99mTc-HMPAO-SPECT/CT and 18F-FDG PET/CT. Despite the fact that magnetic resonance imaging (MRI) and computed tomography (CT) offer high-resolution cardiac anatomical information, their application in patients with CDRIE is hindered by numerous artifacts caused by mechanical devices, including prosthetic valves, peacemakers/ICDs/CRTs generators, electrodes, etc. that profoundly worsen the image quality of CT and MRI. Moreover, some devices are not compatible with MRI [6]. The aim of this systematic review is to provide evidence-based data on the diagnostic value of 99mTc-HMPAO-SPECT/CT and 18F-FDG PET/CT in the context of CDRIE evaluation.

## 2. Materials and Methods

We performed a systematic literature review to critically appraise the evidence for the diagnostic performance of 99mTc-HMPAO-SPECT/CT and 18F-FDG PET/CT in CDRIE assessment. This systematic review was performed in line with the PRISMA 2020 statement [11]. The analysis included articles from PubMed, Embase and Cochrane databases written in English. There was no limit regarding the date of publication. We included studies in adults that presented original data. Search terms were defined as “positron emission tomography”, or “PET” or “SPECT” or “scintigraphy”, accompanied by one of the following: “endocarditis” or “CIED infection” or “device infection” or “CDRIE”. Despite the fact that autologous white blood cells can be radiolabeled using 111In-oxine, this technique was not included in the presented systematic review due to its clear limitations in CDRIE assessment (poorer image quality and significantly higher radiation dose).

After the removal of duplicates, we screened articles based on their titles and abstracts. We rejected case reports, editorials, reviews, abstracts, and studies that did not provide data relevant to the topic of this systematic literature review. Final decisions on the inclusion of references were subsequently done with the consensus of three reviewers. Selected reports were assessed for eligibility based on the full text, the context of the study design, the topic, and the study population. We excluded studies featuring a mixed group of patients with different types of infective endocarditis. After the selection of all the relevant articles, we assessed their methodological quality. The GRADE (Grading of Recommendations Assessment, Development and Evaluation) criteria were applied to evaluate the studies included for any possible bias [11].

## 3. Results

**Figure 1:**
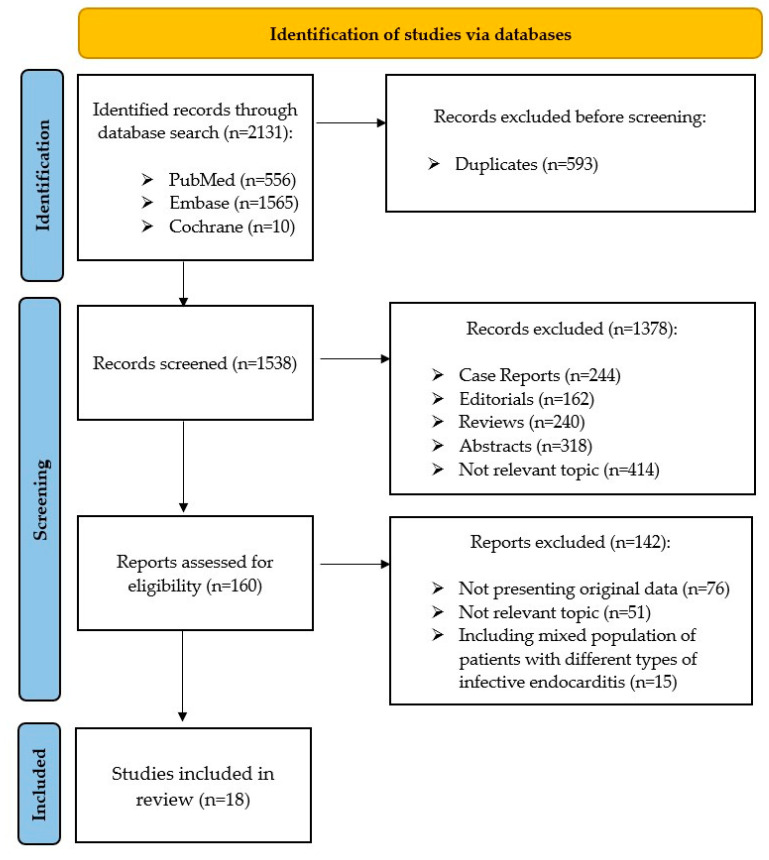
Flow diagram presenting the systematic review, which included searches of databases adhering to the PRISMA 2020 statement. Initially, 2131 records were identified, published between April 1971 and March 2021. Finally, 18 studies were included in the systematic review that presented original data on the diagnostic value of 99mTc-HMPAO-SPECT/CT or 18F-FDG PET/CT in CDRIE. From: Page, M.J.; McKenzie, J.E.; Bossuyt, P.M. et al. The PRISMA 2020 statement: an updated guideline for reporting systematic reviews. BMJ 2021, 372, n71; DOI: 10.1136/bmj.n71 [11]. 18F-FDG PET/CT—positron emission tomography with fluorodeoxyglucose. 99mTc-HMPAO-SPECT/CT—single photon emission tomography and computed tomography with technetium99m-hexamethylpropyleneamine oxime–labeled autologous leukocytes. CDRIE—cardiac device-related infective endocarditis.

Initially, 2131 records published between April 1971 and March 2021 were identified. Based on the analysis, which was performed in accordance with PRISMA criteria, 18 studies that presented original data on the diagnostic value of 99mTc-HMPAO-SPECT/CT or 18F-FDG-PET/CT in CDRIE were included in our systematic review (Figure 1, Table 1) [11]. A single study was identified which compared the diagnostic properties of both modalities directly [12]. Overall, five studies addressed the value of 99mTc-HMPAO-SPECT/CT in cardiac device-related infections [12,13,14,15,16]. The following 14 articles which were incorporated into this systematic review evaluated the diagnostic properties of 18F-FDG PET/CT in CDRIE diagnosis [12,17,18,19,20,21,22,23,24,25,26,27,28,29]. All the studies included met the GRADE criteria for, at best, low quality [30]. Finally, the systematic review encompassed 1094 scans, including 737 cases of 18F-FDG PET/CT and 357 of 99mTc-HMPAO-SPECT/CT.

Most of presented studies included evaluation of extracardiac foci of increased tracer uptake, which were detected up to 34% of patients [12,13,14,15,16,17,18,19,20,21,22,25,26,27]. Besides pulmonary emboli, the remaining detected extracardiac inflammatory foci in the 99mTc-HMPAO-SPECT/CT and 18F-FDG PET/CT were diagnosed as primary sites leading to CDRIE or other non-related concomitant inflammatory lesions, such as neoplastic sites.

### 3.1. Direct Comparison of 99mTc-HMPAO-SPECT/CT and 18F-FDG PET/CT

We included a retrospective cohort study directly addressing the diagnostic value of both 99mTc-HMPAO-SPECT/CT and 18F-FDG PET/CT in suspected CIED infection [12]. The study enrolled 48 patients who underwent both tests within a 30-day time period. The diagnostic sensitivity, specificity, positive predictive value (PPV) and negative predictive value (NPV) were respectively 80%, 91%, 80% and 91% for 18F-FDG PET/CT and 60%, 100%, 100% and 85% for 99mTc-HMPAO-SPECT/CT. The authors concluded that the addition of a positive nuclear imaging result as a major criterion markedly improves the Duke-Li classification at admission, particularly when the infection is initially graded as possible. The study noted that antibiotic therapy might impact the diagnostic properties of these techniques.

### 3.2. 99mTc-HMPAO-SPECT/CT

Overall, there were three prospective studies and two retrospective studies evaluating diagnostic properties of white blood cell scintigraphy in the course of CDRIE [12,13,14,15,16]. All of them assessed patients with suspected CDRIE; however, none included a control group [13,14,15,16]. One study was based on a heterogeneous group consisting of patients who underwent a SPECT/CT scan using two different tracers—Scintimun^®^ (Cisbio, Codolet, France) and 99mTc-HMPAO (GE Healthcare Ltd., Amersham, UK) [14]. In this study, the SPECT-CT had 73.7% sensitivity, 81.0% specificity, 77.5% accuracy, 77.8% PPV and 77.3% NPV; however, these results are based on a non-uniform imaging methodology including scans with two radiotracers and thus should be interpreted with caution.

The following studies included patients who underwent 99mTc-HMPAO-SPECT/CT [12,13,15,16]. This modality was characterized cumulatively by diagnostic properties in the range of 60–93.7% for sensitivity, 88–100% specificity, 84.6–93.9% NPV and 74–100% PPV. Moreover, scintigraphy reliably excluded device-associated infection during a febrile episode and sepsis, with 95% NPV [13]. Inclusion of 99mTc-HMPAO-SPECT/CT into the modified Duke criteria yields significantly higher sensitivity (87% vs. 48%, *p* < 0.001) [15]. Not only did this technique prove highly efficacious in terms of its diagnostic properties, but it also determined the extent of device involvement and detected associated complications. In patients with suspected CDRIE, positive 99mTc-HMPAO-SPECT/CT results are associated with increased rates of in-hospital mortality (11.4% vs. 0%, respectively; odds ratio: 19.6; 95% confidence interval [CI]: 1.02 to 374.70), an increased rate of complications (43% vs. 9%, respectively; hazard ratio [HR]: 5.9; 95% CI: 2.27 to 15.20) and occurrence of a hardware removal procedure (57% vs. 16%, respectively; HR: 4.3; 95% CI: 2.07 to 19.08) [16].

### 3.3. 18F-FDG PET/CT

Based on the analysis performed, we identified 10 prospective and 4 retrospective studies evaluating the diagnostic properties of 18F-FDG PET/CT in CDRIE diagnosis [12,17,18,19,20,21,22,23,24,25,26,27,28,29]. Eight of them included a control group [17,20,22,23,24,26,28,29]. This modality was characterized cumulatively by 86.67–93% accuracy, 30.8–100% sensitivity, 62.5–100% specificity, 66–100% PPV and 75–100% NPV for detection of endocarditis associated with CIED. However, this technique displayed 86.6% accuracy, 72.2–84.2% sensitivity, 95.6–100% specificity, 86.7–94.1% PPV and 88.9–89.6% NPV when detecting local device infection (LDI), which is by definition limited to the pocket and does not involve leads.

Interestingly, two prospective studies reported surprisingly low sensitivity values for this modality in the detection of CDRIE—38.5% and 30.8%, respectively [19,21]. The first study included 63 patients and involved the microbiological evaluation of extracted material [19]. The authors did not find an association between false negative imaging results and the vegetation size, prior antibiotic treatment duration, time between 18F-FDG PET/CT and device extraction, or systemic inflammatory activity. They concluded that the yield of 18F-FDG PET/CT for suspected CIED infections differs depending on the site of infection and that negative studies must be interpreted with caution if the suspicion of CDRIE is high [19]. The latter study enrolled only 21 patients; however, the authors’ conclusions were partially in concordance with the previous study, suggesting that the reliability of 18F-FDG PET/CT findings varies according to the type of CIED infection and is higher in LDI than CDRIE [21]. Moreover, most false-negative results occurred in patients who had undergone previous antimicrobial treatment, which was highlighted in other studies as well [12,21,24,25]. In addition, inclusion of 18F-FDG PET/CT as a major criterion markedly improved the Duke-Li classification at admission [12]. Currently, there is an unmet clinical need for more data supporting the prognostic role of this technique. A single prospective study showed that identification of patients with a “Cold Closed Pocket”, defined as absence of any pocket skin lesion or increased tracer uptake within pocket region at 18F-FDG PET/CT, may be clinically relevant, since this subset of patients present worse long-term survival [25].

The diagnostic performance of this modality is dependent on the proper suppression of background activity from physiological 18F-FDG myocardial uptake by means of a low-carbohydrate high-fat diet and/or fasting. Several of the analyzed studies included solely a 4–12-h long fasting period prior to the study [12,18,19,23,24,26,28,29]. However, in some cases the protocol included a combination of low-carbohydrate diet for 24–48 h before the PET/CT and fasting at least 8–15 h before the study [17,20,21,22,25]. Moreover, some centers did report monitoring blood glucose levels prior to injection of the 18F-FDG tracer, which were within the range 3.5–12 mmol/L [17,19,20,22,26]. Overall, the most prevalent causative pathogens were Staphylococcus aureus and Coagulase-negative Staphylococci [12,17,18,19,20,21,22,23,24,25,26,27,28,29]. None of the conducted studies were particularly designed to account for the potential interference of the type of causative pathogen and accuracy of 18F-FDG PET/CT in CDRIE evaluation. There were reported false negative imaging results in the course of Candida glabrata and Staphylococcus epidermidis infection [12,17,18].

Several studies included evaluation of the maximum standardized uptake values (SUVmax) [12,17,19,20,21,22,23,24,25,26,29]. The SUVmax of the pocket area was significantly higher in patients with CIED infection than in the control group (4.8 ± 2.4 vs. 2.0 ± 0.8, *p* < 0.01) [17]. In patients with confirmed LDI, the involved sides showed greater and heterogeneous uptake, with average uptake values significantly higher than in controls (respectively, SUVmax = 4.72 ± 1.68 and 1.70 ± 0.52; *p* < 0.01) [24]. Because of the individual variability in tracer uptake and in circulating tracer, quantification was not useful for positive diagnosis of lead infection [24]. Importantly, mean lead SUVmax significantly increased on 3-h imaging compared to standard acquisition in patients with infected leads (3.25 ± 0.93 vs. 1.11 ± 1.70, *p* = 0.01) [22].

## 4. Discussion

In recent decades, despite profound advances in microbiological testing and multimodality imaging, the mortality rates associated with IE have not substantially diminished [31]. Furthermore, due to demographic changes and the development of indications for CIED implantation, a relative increase in the population of patients at risk of CDRIE has been seen [32]. Due to the various causative microorganisms, biofilm formation and numerous comorbidities, this disease may have a nonspecific clinical presentation, which hinders proper diagnosis [33]. What is more, differentiation between (i) CDRIE, involving cardiac tissues and/or the intravascular portion of the lead, and (ii) LDI, restricted solely to the CIED lodge, is of paramount importance, since both have different courses and preferred treatments. Infection of the cardiovascular system leads to systemic inflammatory syndrome (SIRS), heart failure, severe complications and death [2]. In fact, patients who suffer a CIED infection are at risk of 15–20% excess absolute mortality after 1 year, and this increased mortality is observed in the group for up to 3 years [34]. Treatment of CDRIE requires a prolonged course of intravenous antibiotic therapy during a hospital stay and complete hardware removal [2]. Indeed, infectious complications are the leading indication for transvenous lead extraction in Europe [4]. Currently, there is no single reliable test confirming diagnosis based on clinical criteria. However, Duke criteria have low diagnostic value, due to high rates of negative microbiological testing and difficulties in interpreting echocardiographic and radiological images with artifacts related to the CIED [2]. Delayed or improper diagnosis may result in detrimental outcomes, such as inappropriate lead extraction or delays in treatment [35]. Hence, strategies aimed at improving the CDRIE diagnostic process could translate into improvements in clinical outcomes.

There has been growing evidence that molecular imaging techniques may provide additional diagnostic value in this complex and challenging group of patients [6,7,8,13,19,23]. However, due to limited reliable data assessing their diagnostic properties, 99mTc-HMPAO-SPECT/CT and 18F-FDG PET/CT were not included in CDRIE diagnostic criteria in European Society of Cardiology (ESC) guidelines and may merely be considered an additive tool in patients with positive blood cultures and non-diagnostic echocardiography [2]. This recommendation was maintained in the recent European Heart Rhythm Association (EHRA) consensus document [36]. Nevertheless, this consensus paper proposed the Novel 2019 International CIED Infection Criteria, which include 99mTc-HMPAO-SPECT/CT and 18F-FDG PET/CT results, which is in line with the results of our systematic review. The analysis performed further expands the available evidence to support the application of nuclear imaging, not only with regards to insights into ongoing infection at a molecular level, but in support of the selection process with respect to potential clinical therapeutic strategies. The inclusion of molecular imaging in the CDRIE diagnostic process could improve the appropriate classification of patients and avoid unnecessary treatment in this group of patients.

The diagnostic profile of the molecular imaging techniques—99mTc-HMPAO-labelled white blood cell scintigraphy and 18F-FDG PET/CT in CDRIE—is presented in Table 2. Since these two modalities are based on different molecular mechanisms, their diagnostic properties, patient preparation, technical limitations and possible interference with ongoing pharmacotherapy differ. The first technique, 18F-FDG PET/CT, is based on evaluating tissue glucose metabolism. Injected 18F-FDG enters the cell through glucose transporters (GLUTs) and is phosphorylated by hexokinases (HXKs) to FDG-6-phosphate, which is then trapped within the cell [37]. Thus, this technique is considered to be very sensitive among nuclear medicine experts and provides quantification opportunities [38]. In addition, it is cost-effective in patients with Gram positive bacteremia and risk factors for septic dissemination, including prosthetic valves [39]. The diagnostic performance of this modality is dependent on the proper suppression of background activity from physiological 18F-FDG myocardial uptake by means of a low-carbohydrate and high-fat diet, followed by an at least 4-h fast [6]. Although the acquisition protocol is short and requires a single scan, this technique is actually quite time-consuming due to the protracted 24-h-long patient preparation time. Moreover, due to the fact that 18F-FDG accumulates in cells with high metabolic activity, a wide range of pathological conditions can mimic IE uptake—vasculitis, primary and metastatic cardiac tumors, postsurgical inflammation and foreign body reactions [6]. Interestingly, these post-surgical reactions were the source of false positive results, mostly in patients with a prosthetic heart valve, as a result of the application of a surgical adhesive [2]. Clearly, these variables should be taken into account when interpreting 18F-FDG PET/CT results.

The second modality, 99mTc-HMPAO-SPECT/CT, relies on the intracellular labeling of autologous leukocytes, which are isolated from whole blood [40]. The tracer and radioisotope 99mTc-HMPAO complex, being lipid-soluble, penetrates the cell membrane of the leukocytes via passive diffusion. Two mechanisms responsible for the retention of 99mTc-HMPAO inside the cell have been suggested: conversion of the complex into a hydrophilic form by reducing agents such as glutathione and binding to non-diffusible proteins and cell organelles [41]. The recent introduction and validation of disposable sterile closed devices for leucocyte labelling has simplified the labelling procedure; still, it remains time consuming and requires blood handling [42]. After intravenous injection, radiolabeled white blood cells migrate to the lungs and, if not damaged, proceed to the liver, the spleen and the reticuloendothelial system, including bone marrow [40]. Further migration, taking place 1 h post injection, is directed to the bone marrow and infected tissues, as a result of chemotactic attraction caused by the presence of biofilm and its soluble products [40]. Thus, 99mTc-HMPAO-SPECT/CT relies on a 24-h-long protocol, including tracer injection, and follow-up early (30–60 min), delayed (2–4 h) and late (20–24 h) acquisitions [6]. The accumulation may be influenced by the virulence and extent of infection, type of pathogen, antibiotic therapy and vascularization of the infected tissue [40]. Nevertheless, this technique provides high specificity, especially in the context of differentiating sterile and infectious morphological intracardiac lesions [43]. These properties should be taken into consideration when interpreting 99mTc-HMPAO-SPECT/CT results.

Importantly, 18F-FDG PET/CT and 99mTc-HMPAO-SPECT/CT provide incremental diagnostic value in implications beyond CDRIE, namely prosthetic valve endocarditis (PVE) and vascular graft infection [2,44]. According to the recent ESC guidelines those modalities are included in the diagnostic criteria and should be used when the diagnosis of PVE remains only ‘possible’ or even ‘rejected’ but with a persisting high level of clinical suspicion [2]. Published data suggested that visual grading score and early imaging prior to antimicrobial treatment may further improve the diagnostic accuracy of 18F-FDG PET/CT [45]. Hopefully in the future, novel diagnostic scores may help to differentiate foreign body reactions and bacterial targeting tracers will be introduced to clinical care [46].

Some of the studies included are classified as low quality according to GRADE criteria. This stems from the low numbers of study participants enrolled, the lack of control groups and their retrospective character. The systematic review carried out is also limited by the heterogeneity of the studies included, with regards to the gold standard and imaging protocol used. Based on analyzed data it seems that it may be especially important, in the context of the proper protocol for suppression of background activity from physiological 18F-FDG myocardial uptake. Presented studies were characterized by heterogenous preparation techniques prior to the tracer injection and reported glucose levels were in a wide range. Proper suppression of myocardial activity is pivotal for optimizing 18F-FDG PET/CT imaging [6]. Thus, those different myocardial suppression protocols should be considered in the context of discrepancies in 18F-FDG PET/CT diagnostic performance in CDRIE assessment. The results of this systematic analysis show that both 18F-FDG PET/CT and 99mTc-HMPAO-SPECT/CT provide additional diagnostic value in CDRIE work-up. However, the data presented is limited to mostly small studies; in the future these results should be validated in larger multi-center trials. There are substantial discrepancies with respect to the reported sensitivity of 18F-FDG PET/CT for detection of endocarditis associated with CIED, which might be a result of choosing various gold standards as diagnostic reference (Table 2) and the myocardial suppression protocol used.

Future research directions should verify whether these techniques may be used to monitor antimicrobial CDRIE treatment and the guide time of the complete hardware removal procedure. Serial assessment of the inflammatory status using 18F-FDG PET/CT might be helpful for monitoring therapy efficacy and for deciding treatment continuation, tapering or change of treatment in cardiac sarcoidosis and vascular graft infection [47,48]. It should be investigated whether there is pathogen variability in 99mTc-HMPAO-labelled white blood cell scintigraphy and 18F-FDG PET/CT results. Clearly, there is a need for larger, prospective, multicenter trials to further develop molecular imaging in the field of cardiovascular infections.

## 5. Conclusions

Appropriate CDRIE diagnosis remains a challenge due to complex etiopathogenesis and clinical presentation. Nuclear medicine imaging modalities provide additional insight into ongoing CIED-associated infections. The differing underlying molecular mechanisms of 99mTc-HMPAO-SPECT/CT and 18F-FDG PET/CT translate to various technical properties and limitations, which should be considered when interpreting scans. Notwithstanding this, both modalities provide additional diagnostic value in the identification of patients at risk of CDRIE and should be considered in the CDRIE diagnostic process. Based on the evidence presented here, it seems that the inclusion of positive imaging results derived from these techniques, utilized as major diagnostic criteria (alongside the standard work-up), helps to classify patients more effectively [12,15]. Moreover, 99mTc-HMPAO-SPECT/CT identifies patients at higher risk of in-hospital death, complications and a complete hardware removal procedure [16]. Furthermore, a selected finding in 18F-FDG PET/CT (‘Cold Closed Pocket’) is associated with all-cause mortality [25]. These results further expand the evidence to support the application of nuclear imaging, not only for the insights gained into ongoing infection at a molecular level, but also for the determination of potential clinical therapeutic strategies. Ultimately, the inclusion of molecular imaging into the CDRIE diagnostic process might improve prognosis and avoid unnecessary treatment in this group of patients.

## Figures and Tables

**Table 1:** Studies evaluating the diagnostic value of 99mTc-HMPAO-labelled white blood cell scintigraphy and 18F-FDG PET/CT in cardiac device-related infective endocarditis [12,13,14,15,16,17,18,19,20,21,22,23,24,25,26,27,28,29].

Study	Inclusion	Number of Cases and Controls	Gold Standard	Diagnostic Accuracy ^1^	Quality ^2^
18F-FDG PET/CT (n = 737, 539 cases and 198 controls)					
Rodríguez-Alfonso, B.	retrospective	44	expert multidisciplinary team	sensitivity 84.0% (65.3–93.6%), specificity 94.7% (75.4–99.1%), PPV 95.5% (78.2–99.2%), NPV 81.8% (65.7–97.9%); LDI: sensitivity 84.2% (62.4–94.5%), specificity 96% (80.5–99.3%), PPV 94.1% (73–99%), NPV 88.9% (71.9–96.1%)	low
Ahmed, F.Z.	prospective	46 and 40 controls	lead cultures, clinical assessment by an experienced cardiologist	group which underwent extraction (n = 32): sensitivity 97% (84–100%), specificity 98% (90–100%); group treated conservatively (n = 14) sensitivity 76% (61–87%), specificity 100% (87–100%)	high
Ploux, S.	prospective	10 and 40 controls	lead cultures and 12-month follow up	sensitivity 100%, specificity 93%, PPV 66%, NPV 100%	low
Graziosi, M.	prospective	27	expert multidisciplinary team, modified Duke criteria, 6-month follow-up	sensitivity 63%, specificity 86%, PPV 77%, NPV 76%	low
Tlili, G.	retrospective	40 and 40 controls	clinical evaluation, lead cultures and 12-month follow up	accuracy 90%, sensitivity 83%, specificity 95%, PPV 94%, NPV 88%	low
Diemberger, I.	prospective	105	expert multidisciplinary team, modified Duke criteria, 6–12-month follow-up	sensitivity 91.4% ‘Cold Closed Pocket’ associated with mortality: HR 2.84 (1.37–5.89; *p* = 0.005)	high
Bensimhon, L.	prospective	21 and 14 controls	lead cultures, modified Duke criteria, 6-month follow-up	accuracy 90.4%, sensitivity 80%, specificity 100%, PPV 100%, NPV 84.6%	low
Sarrazin, J.F.	prospective	42 and 24 controls	Duke criteria, conventional tests	sensitivity 88.6% (72.3–96.3%), specificity 85.7% (42–99.2%)	high
Leccisotti, L.	prospective	27 and 15 controls	bacteriological analysis, follow-up (at least 3 months) for control group	accuracy 93% (76–99%), sensitivity 86% (65–97%), specificity 100% (48–100%) delayed 18F-FDG PET/CT: accuracy 91% (71–99%), sensitivity 91% (71–99%), specificity 100% (48–100%)	high
Jerónimo, A.	prospective	63	clinical data, lead cultures, TEE, pulmonary embolisms detected by PET/CT	sensitivity 38.5% (11.7–64.5%), specificity 98% (89.5–99.6%), PPV 83.3% (43.6–97%), NPV 86% (74.7–92.7%); LDI: sensitivity 72.2% (49.1–87.5%), specificity 95.6% (85.2–98.8%), PPV 86.7% (62.1–96.3%), NPV 89.6% (77.8–94.5%)	high
Calais, J.	retrospective	48	multidisciplinary expert team, lead cultures, Duke-Li criteria, at least 3-month follow-up	sensitivity 80% (51.9–95.7%), specificity 90.9% (75.7–98.1%), PPV 80% (56.9–92.4%), NPV 90.9%, (78.3–96.5%); diagnostic criteria including 18F-FDG PET/CT: sensitivity 100.0% (78.2–100%), specificity 84.9% (68.1–94.9%), PPV 75.0% (57.2–87.1%), NPV 100%	high
Salomäki, S.P.	prospective	30 and 10 controls	modified Duke Criteria, follow-up	sensitivity 90%, specificity 73%, PPV 75%, NPV 89%	low
Cautela, J.	prospective	21	lead cultures, Duke criteria	sensitivity 30.8% (9.1–61.4%), specificity 62.5% (24.5–91.5%); LDI: sensitivity 86.7% (59.5–98.3%), specificity 100% (42.1–100%)	low
Rubini, G.	retrospective	15 and 15 controls	clinical guidelines, lead cultures	accuracy 86.67% (59.54–98.34%), sensitivity 90.91% (58.72–99.77%), specificity 75% (19.41–99.37%), PPV 90.91% (64.45–98.22%), NPV 75% (29.86–95.48%)	low
**99mTc-HMPAO-SPECT/CT (n = 357)**					
Calais, J.	retrospective	48	multidisciplinary expert team, lead cultures, Duke-Li criteria, at least 3-month follow-up	sensitivity 60% (32.3–83.7%), specificity 100% (89.4–100%), PPV 100%, NPV 84.6% (74.7–91.1%) diagnostic criteria including 99mTc-HMPAO-SPECT/CT: sensitivity 93.3% (68.1–99.8%), specificity 90.9% (75.7–98.1%), PPV 82.4% (61.1–93.3%), NPV 96.8% (81.8–99.5%)	high
Małecka, B.	prospective	40	modified Duke criteria	sensitivity 73.7% (55.1–86.1%), specificity 81% (64.2–92.2%), PPV 77.8% (58.2–90.9%), NPV 77.3% (61.3–88%)	low
Holcman, K.	prospective	103	multidisciplinary expert team, lead cultures, 6-month follow-up including outpatient visit with TTE	accuracy 86% (78–92%), sensitivity 84% (71–97%), specificity 88% (80–95%), NPV 93% (86–99%), PPV 74% (60–89%) diagnostic criteria including 99mTc-HMPAO-SPECT/CT: accuracy 88% (81–94%), sensitivity 87% (75–99%), specificity 89% (82–96%), NPV 94% (89–100%), PPV 77% (63–91%)	high
Erba, P. A.	retrospective	63	lead cultures, Duke criteria, follow-up	accuracy 96.8% (88–99.4%), sensitivity 93.7% (83.9–98%), specificity 100% (92.8–100%), PPV 100% (92.8–100%), NPV 93.9% (84.1–98.1%)	high
Holcman, K.	prospective	103	multidisciplinary expert team, lead cultures, 17.48 ± 11.90-month follow-up including outpatient visit with TTE	in-hospital mortality OR 19.6 (1.02–374.3), complications HR 5.9 (2.27–15.2), complete hardware removal HR 4.3 2.07–19.08	high

^1^ Values presented with 95% confidence intervals. ^2^ Quality rated according to the GRADE approach [30]. Abbreviations are listed in the Figure 1 legend. HR—hazard ratio, LDI—local device infection, NPV—negative predictive value, OR—odds ratio, PPV—positive predictive value, TEE—transesophageal echocardiography, TTE—transthoracic echocardiography.

**Table 2:** Diagnostic profile of molecular imaging techniques—99mTc-HMPAO-labelled white blood cell scintigraphy and 18F-FDG PET/CT in cardiac device-associated infections.

	18F-FDG PET/CT	99mTc-HMPAO-SPECT/CT
**Patient preparation**	at least 4 h fasting, 24 h low-carbohydrate and high-fat diet, in some protocols intravenous heparin or calcium channel blockers are administered prior to the acquisition	tracer preparation involving isolation of autologous leucocytes, incubation with tracer HMPAO and radioisotope 99mTc, followed by intravenous autologous radiolabeled leucocytes administration
**Duration**	Short—single acquisition 60 min after tracer injection	Long—24 h, including tracer injection and early, delayed and late acquisitions
**Radiation dose**	2.5–5.0 Megabecquerels/kilogram, that is 175–350 Megabecquerels in a 70-kg standard adult	370–740 Megabecquerels
**Quantification**	possible	limited
**Anatomical resolution**	good	sufficient
**Availability**	different across countries	different across countries
**Diagnostic properties** **-direct comparison** **-cumulative data**	-more sensitive 30.8–100% sensitivity, 62.5–100% specificity, 75–100% NPV, 66–100% PPV	-more specific 60–93.7% sensitivity, 88–100% specificity, 85–93.9% NPV, 74–100% PPV
**Prognostic value**	‘Cold Closed Pocket’ is associated with mortality	positive result for CDRIE is associated with increased in-hospital mortality and complication rates
**Quantity of supporting literature evidence**	moderate	limited
**Limitations**	- myocardial and respiratory artifacts - inflammatory lesions difficult to differentiate from infection sites - limited detection of smaller vegetations along CIED leads- better properties for diagnosing LDI than CDRIE	- metallic artifacts - non-specific activity in the bowel as a result of hepatic HMPAO excretion - limited detection of smaller vegetations
**Interference from ongoing steroid treatment**	probable	no evidence
**Interference from ongoing antimicrobial treatment**	probable	probable
**Contraindications**	-pregnancy -uncontrolled diabetes mellites	-pregnancy -neutropenia

Abbreviations are listed in the Figure 1 and Table 1 legends. HMPAO—hexamethylpropyleneamine-oxime.
